# A Photoacoustic Imaging Algorithm Based on Regularized Smoothed L_0_ Norm Minimization

**DOI:** 10.1155/2021/6689194

**Published:** 2021-06-01

**Authors:** Xueyan Liu, Limei Zhang, Yining Zhang, Lishan Qiao

**Affiliations:** Department of Mathematics Science, Liaocheng University, Shandong 252000, China

## Abstract

The recently emerging technique of sparse reconstruction has received much attention in the field of photoacoustic imaging (PAI). Compressed sensing (CS) has large potential in efficiently reconstructing high-quality PAI images with sparse sampling signal. In this article, we propose a CS-based error-tolerant regularized smooth L0 (ReSL0) algorithm for PAI image reconstruction, which has the same computational advantages as the SL0 algorithm while having a higher degree of immunity to inaccuracy caused by noise. In order to evaluate the performance of the ReSL0 algorithm, we reconstruct the simulated dataset obtained from three phantoms. In addition, a real experimental dataset from agar phantom is also used to verify the effectiveness of the ReSL0 algorithm. Compared to three L_0_ norm, L_1_ norm, and TV norm-based CS algorithms for signal recovery and image reconstruction, experiments demonstrated that the ReSL0 algorithm provides a good balance between the quality and efficiency of reconstructions. Furthermore, the PSNR of the reconstructed image calculated by the introduced method was better than the other three methods. In particular, it can notably improve reconstruction quality in the case of noisy measurement.

## 1. Introduction

Photoacoustic imaging (PAI) is a new noninvasive and nonionizing biomedical imaging method which gains rapid development in the last two decades [1–4]. As a hybrid technique, PAI attains the advantages of the contrast of optical imaging and high resolution of ultrasonic imaging. In particular, PAI has shown great potential in many applications, including living mouse brain vasculature imaging [3, 5, 6], human skin imaging [7, 8], and the treatment and diagnosis of cancer [9–11]. When the biological tissue is irradiated by nanosecond laser pulses, the photoacoustic signals will be generated in the tissue's light absorption region and measured by ultrasonic transducers. Afterwards, the distribution of laser energy absorption can be calculated from the photoacoustic signals by using the analytical or iterative algorithms.

The reconstruction algorithm is an important factor affecting the quality of PAI imaging, and the accurate and efficient reconstruction algorithms are of great significance. Analytic algorithms such as the filtered back-projection algorithm [12–14], the deconvolution reconstruction algorithm [15], and the time-reversal imaging algorithm [16] have already been used extensively owing to their accuracy and implementation convenience. However, the analytic algorithms can only reconstruct an accurate image with complete measurement data from all directions, which requires a long scan time or complex electronic equipment. When the data are insufficient, the analytic algorithms are no longer effective. In many PAI applications, such as breast imaging and ophthalmic imaging, only incomplete data can be accepted. There has not been accurate PAI reconstruction formula with incomplete data yet. Therefore, the development of high-speed and high-quality PAI image reconstruction algorithms based on incomplete data is an active research topic recently. The incomplete data may arise from various forms, but in this work, we only consider the sparse-view PAI reconstruction problem.

Mathematically, image reconstruction with sparse-view incomplete data can be thought of as an underdetermined linear system. By making some constraints, the iterative reconstruction algorithm that gives a more accurate result at the expense of much more computing time has been developed for sparse-view PAI [17–19]. One type of them is based on the compressive sensing (CS) theory, which has drawn increasing attention due to its can recover sparse signals under sampling rate far lower than the Nyquist rate [20, 21]. By using the L1magic convex optimization algorithm and sparse-view data, Provost and Lesage applied CS to PAI [22, 23]. The problem of artifacts and loss of resolution in the case of insufficient measurements can be solved by using patterned excitation light via the SPGL1 algorithm [24]. Bayesian CS method was used to simplify the PAI system [25]. The experimental results indicated that the CS-based reconstruction method can reduce undersampling artifacts effectively through the nonlinear conjugate gradient descent algorithm [26]. The above studies indicated that the CS methods can reduce the number of ultrasonic transducers and get high-quality reconstruction results with sparse-view data.

To date, most CS applications within sparse-view PAI imaging have centered on the L_1_ norm minimization problem [22, 24, 27] and the total variation (TV) minimization problem [28–31]. Encouraged by the advantages in edge preservation and finer structure recovering, the L_0_ regularizations were introduced to computed tomography (CT) [32–35], magnetic resonance imaging (MRI) [36], and PAI [37]. Bioucas-Dias and Figueiredo proposed a smoothed L_0_ norm (SL0) [38] algorithm that directly minimizes the L_0_ norm, which combines the advantages of the high precision of convex optimization and the rapidity of greedy algorithm. Mozaffarzadeh et al. have shown that the SL0 algorithm can provide a higher PAI image quality while a low number of transducers were [37]. However, the SL0 algorithm does not consider the noise in the measurement data. Based on the SL0 algorithm, Bu et al. have adopted a regularization term to tolerate errors which can achieve the same computational efficiency as the SL0 algorithm while having better noise robustness. This inspired us to apply the regularized smooth L_0_ (ReSL0) algorithm to reconstruct the sparse-view PAI image. In this paper, we studied the use of the ReSL0 for L_0_ norm minimization CS problems caused by sparse-view PAI reconstruction. The proposed algorithm is able to provide more accurate result under the sparse-view situation. For the purpose of verifying the capability of the ReSL0 algorithm, the ReSL0 algorithm compares with three L_0_ norm, L_1_ norm, and TV norm-based CS algorithms for signal recovery and image reconstruction.

## 2. Methods

### 2.1. Photoacoustic Theory

Based on the photoacoustic signal generation theory, the relationship between the acoustic pressure *p*(**r**, *t*) and the absorbed energy density *A*(**r**) obeys the following wave equation [39].

(1) $$\begin{array}{c} \left(\frac{\partial^{2}}{\partial t^{2}}-c^{2}\nabla^{2}\right)p\left(r,t\right)=\Gamma \left(r\right)A\left(r\right)\frac{\partial I\left(t\right)}{\partial t}, \end{array}$$

where **r** means the pixel position, *t* indicates the time, *c* is the ultrasound speed, Γ(**r**) denotes the Grueneisen parameter, and *I*(*t*) is a temporal function of illumination that can be approximately regarded as a Dirac delta function *δ*(*t*) in most practical cases. By solving the wave equation of Eq. (1), the pressure measured at locations **r**_0_ can be written as [40].

(2) $$\begin{array}{c} p\left(r_{0},t\right)=\frac{\Gamma \left(r\right)}{4\pi c^{2}}∭\frac{A\left(r\right)}{\;|\;r-r_{0}\;|\;}\delta \left(t-\frac{\;|\;r-r_{0}\;|\;}{c}\right)d^{3}r. \end{array}$$

With Fourier transform, the forward projection problem can be expressed in the time-frequency domain as

(3) $$\begin{array}{c} \bar{p}\left(r_{0},k\right)=\frac{ik\Gamma }{4\pi c}∭A\left(r\right)\frac{\exp\left(-ik\;|\;r_{0}-r\;|\;\right)}{\;|\;r_{0}-r\;|\;}d^{3}r. \end{array}$$

During the experiment, the frequency domain data can be obtained by applying fast Fourier transform to the time domain measurements. To numerically model the above forward problem, the unknown reconstruction image *A*(**r**) is reshaped into one-dimensional long vector *X* ∈ *R*^*N*^, and a column vector *Y* ∈ *R*^*M*^ can be used to represent all the temporal-frequency pressure $\bar{p}\left(r_{0},k\right)$. According to Eq. (3), the temporal-frequency domain measurement matrix can be designed as [22].

(4) $$\begin{array}{c} K_{\left(m,n\right)\left(i,j\right)}={ick}_{n}\frac{e^{-{ik}_{n}\;|\;r_{m}-r_{ij}\;|\;}}{\;|\;r_{m}-r_{ij}\;|\;},m=1,2,\cdots ,p,n=1,2,\cdots ,q, \end{array}$$

which is determined by the geometry and the grid shape of the unknown image. In Eq. (4), *r*_*m*_ indicates the position of the transducer, *r*_*ij*_ denotes the pixel coordinates of the image, *p* is the number of ultrasonic sensors, and *q* indicates the number of sampling points, respectively. Then, Eq. (3) can be expressed as *Y* = *KX*. In PAI, the goal of image reconstruction is to reconstruct *X* through the pressure data *Y*.

### 2.2. Compress Sensing Application in PAI

According to the theory of compress sensing, an image can be reconstructed when it or its transformation is sparse. Fortunately, most medical images can be considered sparse with a sparse transform basis Ψ : *X* = Ψ*θ*, where *θ* ∈ *R*^*N*^ indicates the sparse coefficient. Provost and Lesage have shown that there is a sparse transform basis in which the PAI image is compressible [22]. By denoting **A** = **K**Ψ, the problem of PAI image reconstruction can be solved by the following optimization model.

(5) $$\begin{array}{c} \underset{X}{\min}\;E\left(X\right)\;s.t.\;Y=KX=A\theta , \end{array}$$

where *E*(*X*) is the regularization function. Accroding to the literature [22], the matrix *A* obtained from the product between the forward operator *K* in the Fourier domain with a wavelets basis Ψ will be a CS-matrix. And the wavelet basis showed the best properties among generic bases that can represent sparsely images obtained via PAI.

We note that most of the current CS-based reconstruction algorithms explore the prior knowledge that the PAI image is sparse or sparse in the transform domains. And the regularization term in Eq. (5) usually denoted by the L_1_ norm of the image's sparsity transform coefficient, the total variation norm of the image, and so forth. For example, the constrained L_1_ norm minimization can be applied to reconstruct the PAI image.

(6) $$\begin{array}{c} \underset{\theta }{\min}\;{\left\|\theta \right\|}_{1}\;s.t.\;\left\|Y-A\theta \right\|<\delta . \end{array}$$

The subject of solving Eq. (6) has received considerable attention in PAI image reconstruction, such as the use of L1magic [22], SPGL1 [24], and ADM [27].

If TV norm is selected as the regularization term, the TV-based CS reconstruction model can be defined as follows:

(7) $$\begin{array}{c} \underset{X}{\min}\;TV\left(X\right)\;s.t.\;\left\|Y-KX\right\|<\delta , \end{array}$$

where $TV\left(X\right)=\sum_{i=1}^{N}\left\|D_{i}X\right\|=\sum_{i=1}^{N}\sqrt{\Delta_{i}^{h}X^{2}+\Delta_{i}^{v}X^{2}}$ is the discrete form of TV for a grayscale image and Δ_*i*_^*h*^ and Δ_*i*_^*v*^ denote the horizontal and vertical difference operators. In the field of PAI, the TV minimization optimization algorithm can reconstruct excellent image from few-view data [28–31]. In this work, the two-step iterative shrinkage thresholding (TwIST) algorithm is considered for image reconstruction [31, 38].

### 2.3. Regularized Smooth L_0_ Algorithm

In order to realize fast recovery of sparse signal, Mohimani et al. introduced the SL0 algorithm [41] to obtain sparse solution of underdetermined of linear equation *Y* = *KX*. The SL0 algorithm obtains the most sparse solution by solving the following optimization problem

(8) $$\begin{array}{c} \underset{\theta }{\min}\;{\left\|\theta \right\|}_{0}\;s.t.\;Y=A\theta , \end{array}$$

where ‖*θ*‖_0_ is the L_0_ norm of *θ* [37, 41]. In this algorithm, a continuous function was used to approximate ‖*θ*‖_0_ instead of minimize the L_0_ norm directly, which can be written as follows: *f*_*σ*_(*θ*) = exp(−*θ*^2^/*σ*^2^). It should have a parameter *σ* which determines the quality of the approximation. Considering

(9) $$\begin{array}{c} \underset{\sigma \to 0}{\lim}f_{\sigma }\left(\theta \right)=\left\{\begin{array}{cc} 1; & \text{if}\theta =0,\\ 0; & \text{if}\theta \neq 0, \end{array}\right. \end{array}$$

and by defining *F*_*σ*_(*θ*) = ∑_*i*=1_^*N*^*f*_*σ*_(*θ*), the L_0_ norm of *θ* can be calculated by ‖*θ*‖_0_ ≈ *N* − *F*_*σ*_(*θ*) for small values of *σ*.

Recently, Mozaffarzadeh et al. illustrate that SL0 offers higher-quality PAI images in comparison with L_1_ norm-based basis pursuit method while a low number of transducers were [37]. However, we can only observe inaccurate measurements *Y* = *KX* + *e*, which means there are errors between *Y* and *KX*. And the property of the SL0 decreases significantly due to the equality constraint *Y* = *KX*. In order to resolve this problem, we added observation noise to the forward model.

(10) $$\begin{array}{c} Y=KX+e=A\theta +e, \end{array}$$

where *e* ∈ *R*^*M*^ denotes a vector indicating the modeling transducer noise. And a regularized SL0 (ReSL0) method was used to solve the above problem mentioned in Eq. (10) [42, 43], which can be written as follows:

(11) $$\begin{array}{c} \underset{\theta }{\max}\;F_{\sigma }\left(\theta \right)\;s.t.\;{\left\|Y‐A\theta \right\|}_{2}<\delta . \end{array}$$

ReSL0 transforms the equality constraint in Eq. (8) into inequality constraint allowing certain error tolerance. The ReSL0 algorithm includes two nested iterations. The initial value *σ*_0_ is set to 4 times of the maximum absolute value of the sparse coefficients in the outer loop, and the next values *σ*_*j*_ = *ρσ*_*j*−1_, *j* ≥ 1, where *ρ* is chosen between 0.5 and 1 in the experiment [41]. For every *σ*, the internal circle is responsible for finding the maximum value of *F*_*σ*_(*θ*) on set {*θ*|‖*Y*‐*Aθ*‖_2_ < *δ*}. The internal loop consists of iterations of the form *θ* ← *θ* + *μσ*^2^∇*F*_*σ*_(*θ*), followed by solving the optimization problem:

(12) $$\begin{array}{c} \underset{\theta_{p}}{\min}\;\left\|\theta_{p}-\hat{\theta }\right\|\;s.t.\;{\left\|Y‐A\theta \right\|}_{2}<\delta . \end{array}$$

Using Lagrange multipliers, this minimization results in [42].

(13) $$\begin{array}{c} {\hat{\theta }}_{p}=\hat{\theta }-A^{H}{\left({AA}^{H}+\lambda^{-1}I_{M}\right)}^{-1}\left(A\hat{\theta }-y\right), \end{array}$$

where *λ* is the regularized parameter that is fixed for the internal loop and adaptively computed for external loop. An adaptive regularized parameter selection approach [44] is used to solve objective function in Eq. (11), which generates a suitable regularization parameter in the iterative process to balance the fit of the sparsity and residual error in the objective function. Then, the regularized parameter during the internal loop can be written as

(14) $$\begin{array}{c} \lambda =\frac{{\left\|\theta_{\nabla }-{\hat{\theta }}_{j-1}\right\|}_{2}}{{\left\|A^{H}\left(A{\hat{\theta }}_{j-1}-y\right)\right\|}_{2}}, \end{array}$$

where *θ*_∇_ is the first solution of the internal loop for the value *σ* = *σ*_*j*_. The step-by-step procedures of the ReSL0 algorithms are described in Algorithm 1. Other details of the scheme are described in [38, 42, 44] and their references.

## 3. Experiment and Result

Although the existing CS-based PAI reconstruction algorithms provide better results, the accuracy and efficiency of sparse-view PAI reconstruction still need further improvement. In this section, we provided a variety of simulations and in vitro applications to illustrate the advantages and efficiency of the ReSL0 method for sparse-view PAI reconstruction. The forward simulation and inverse reconstruction were conducted in 2D phantoms and images. The same iteration stopping criteria *δ* = ‖*X*^*k*+1^ − *X*^*k*^‖/‖*X*^*k*^‖ < 0.005 were used to be fair to compare the four algorithms. The simulation experiments were carried out using Matlab (version 7.8) on a PC with a 8 GHz CPU and 32 GB memory.

**Figure 1:**
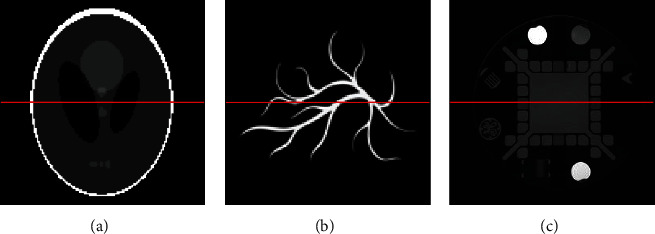
The (a) Sheep-Logan phantom, (b) blood vessel phantom, and (c) standard General Electric resolution phantom employed in the 2D computer-simulation studies.

By using the wavelet transform and different sparse regularization methods, the numerical simulations have been carried out on the Sheep-Logan phantom, the blood vessel phantom, and the standard General Electric resolution phantom (Figure 1) with a 128 × 128 resolution corresponding simulation area of 30 mm × 30 mm. During the simulation, the sound speed was 1500 m/s. A single ultrasonic transducer was used to receive the signals. To simulate the frequency response of the transducer, at every detection position, 128 randomly selected in the window [0.2, 3] MHz were used to define the projection matrix **K**. By adjusting the phantom gray values to [0, 1], we obtained the ultrasonic pressure *Y* using the projection matrix **K**.

### 3.1. Reconstruction from Simulated Sparse-View Data

**Figure 2:**
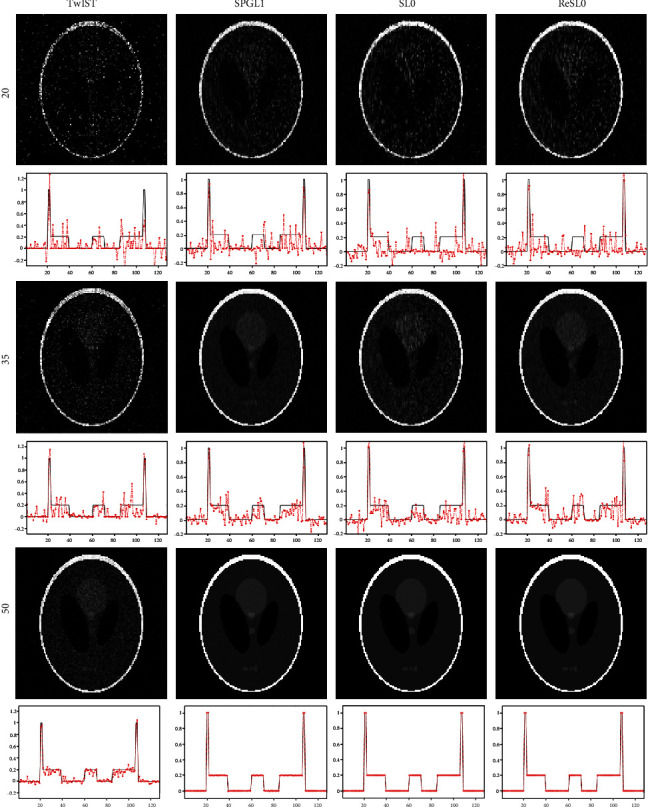
Reconstruction results of the Sheep-Logan phantom. The first to the third rows are the reconstruction images with 20-view, 35-view, and 50-view that are uniformly distributed at a 360° curve. The first to the fourth columns show the results of TwIST, SPGL1, SL0, and ReSL0 individually.

We compared ReSL0 for the solution model (10) with SPGL1 for the solution model (6), TwIST for the solution model (7), and SL0 for the solution model (8).  Figure 2 demonstrates the reconstruction results of the Sheep-Logan phantom by using these four methods. From the first row of Figure 2, we can see that the images of these four methods are strongly affected when 20 position samples are used. When the number of sampling is 35, the images reconstructed by the TwIST and SL0 methods contain artifacts and distortions, and the quality of the reconstructed images by the RESL0 and SPGL1 methods is better than these two methods. As the signal acquisition position reaches 50, the image reconstructed by the TwIST method still contains many noises, and the other three methods can reconstruct high-quality images. So we can conclude that the L_0_ norm and L_1_ norm-based CS algorithms can obtain more accurate image with sparse-view signals.

The quantitative evaluation of the reconstructed images including the CPU times, the peak signal-to-noise ratio (PSNR), and the normalized mean absolute error (NMAE) achieved by each of the algorithms is presented in Table 1. The PSNR (in dB) is defined as $10\log_{10}\left(N\cdot {\mathrm{MAX}}_{I}^{2}/{\left\|X-\hat{X}\right\|}_{2}^{2}\right)$ and the NMAE is defined as $100\times {\left\|\hat{X}-X\right\|}_{2}/{\left\|X\right\|}_{2}$, where MAX_*I*_ means the maximum pixel value of the image and $\hat{X}$ denotes the estimate of the original. The CPU running time was used to estimate time complexity. The PSNR and the NMAE were used for quality evaluation of the reconstructed image.

According to Table 1, we can infer that there are great differences between the CPU times: TwIST is about 1.5 times faster than ReSL0, which itself can be roughly 7 times faster than SPGL1. And the SL0 has the same computational complexity as the ReSL0. From Table 1, we can see that those four methods achieve similar PSNRs and NMAEs when using signals at less than 35 locations. Table 1 also shows that the ReSL0 outperforms other three methods when using signals with more than 35 locations. Moreover, if we consider a method cannot reconstruct a high-quality image as the PSNR is less than 30, then we can conclude that the TwIST fails for all experiments, the SL0 fails when the number of sampling positions is less than 45, while the SPGL1 and ReSL0 algorithms fail when the number of sampling positions is less than 40. The numerical results in Table 1 indicate that the ReSL0 algorithm can reconstruct high-quality images with more than 40 sampling signals in a short time. From the results of simulations, we can see that the ReSL0 algorithm is more accurate than the other three algorithms in the sparse-view sampling condition.

**Figure 3:**
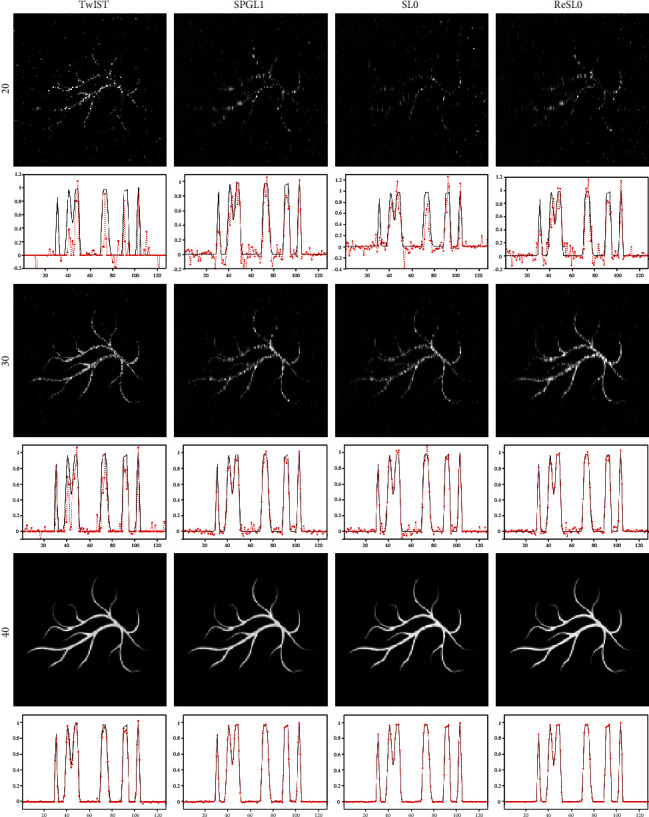
Reconstruction results of the blood vessel phantom. The first to the third rows are the reconstruction images with 20-view, 30-view, and 40-view that are uniformly distributed at a 360° curve. The first to the fourth columns show the results of TwIST, SPGL1, SL0, and ReSL0 individually.

Considering the universality of the ReSL0 method, the blood vessel phantom was used as the initial energy density to additionally compare these four algorithms. The conditions of this experiment were the same as the Sheep-Logan experiment.  Figure 3 shows the reconstruction images of the blood vessel phantom with these four algorithms. It can be seen from Figure 3 that all these four algorithms can reconstruct accurate images by using photoacoustic signals from 40 sampling positions. When the signal of 30 sampling positions is used, all reconstructed images contain much noise, which has a certain influence on the identification of the blood vessel structure. Among these four algorithms, the ReSL0 algorithm has the best reconstruction quality, which can be observed by the middle extraction lines from the reconstructed images. However, when the sampling position is 20, none of the four algorithms can get good reconstructed image.

Table 2 shows the numerical results of the blood vessel for those four methods. Since the reconstructed image sizes of the two experiments are the same, the running time of the blood vessel experiment is similar to that of the Sheep-Logan experiment. From Table 2, we can see that the TwIST method can obtain a larger PSNR and a smaller NMAE when the number of samples is less than 30. However, the NMAE of the TwIST method is only slightly improved when the sampling position is greater than 30. And the ReSL0 method can achieve the largest PSNR and the smallest NMAE when the number of samples is greater than 30. According to Table 2, we can conclude that the TwIST method has better accuracy and efficiency when the signal number is small and the ReSL0 method obtains better quality PAI images with sufficient measurements.

**Figure 4:**
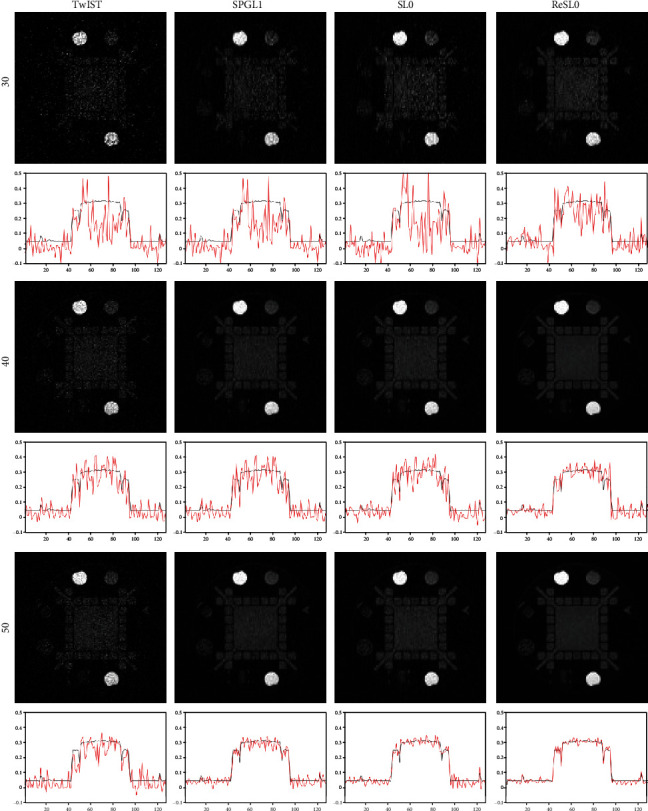
Reconstruction results of the standard General Electric resolution phantom. The first to the third rows are the reconstruction images with 30-view, 40-view, and 50-view that are uniformly distributed at a 360° curve. The first to the fourth columns show the results of TwIST, SPGL1, SL0, and ReSL0 individually.

In order to further verify the effectiveness of the RESL0 algorithm, a more complex and challenging standard General Electric resolution phantom is also used for simulation. The reconstruction results of the four algorithms are shown in Figure 4. In the first column of Figure 4, the images reconstructed by TwIST contain severe noises, which seriously affect the image quality. The second and third columns of Figure 4 are reconstructed by SPGL1 and SL0, respectively. It can be observed that both methods can suppress noise, but the quality of the reconstructed image can be further improved. The last column of Figure 4 shows the results of ReSL0, which obtain the best reconstruction quality among the four methods.

Table 3 shows the numerical results of the standard General Electric resolution phantom for those four methods. As can be seen from Table 3, the TwIST algorithm has the shortest CPU time and the CPU time of the ReSL0 algorithm is slightly slower than the SL0 algorithm, while the CPU time of the SPGL1 algorithm is always much bigger than the other three algorithms. The TwIST algorithm has the worst PSNR and the biggest NMAE, and the PSNR value of images reconstructed by the ReSL0 algorithm is the highest, and the NMAE value of images reconstructed by the ReSL0 algorithm is the lowest. From the above three experiments, we can arrive at the conclusion that the ReSL0 algorithm outperforms the other three algorithms under sparse sampling condition.

### 3.2. Comparison for Different Variances of the Noise

**Figure 5:**
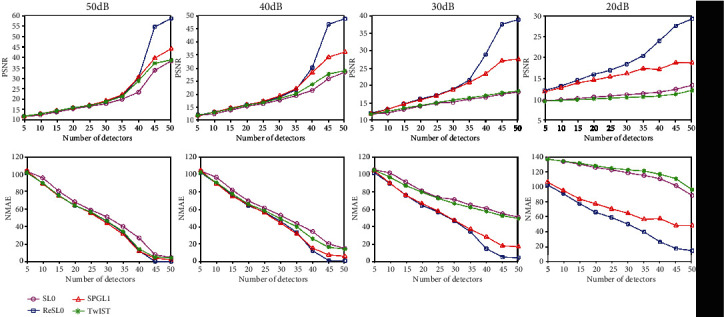
The PSNRs and NMAEs of reconstruction results of the Sheep-Logan phantom with different number of sampling points. (a–d) The quantitative results of noisy observation with SNR = 50, 40, 30, and 20 dB, respectively.

In order to quantify the influence of noise, the PSNR and NMAE of the reconstruction images of the Sheep-Logan phantom experiment are calculated and shown in Figure 5. A good algorithm has a larger PSNR and a smaller NMAE. As can be seen from Figure 5, when there is weak noise of 50 dB and 40 dB, those four methods achieve similar PSNRs and NMAEs when using signals with less than 35 positions. Furthermore, the ReSL0 algorithm achieves the biggest PSNR and the smallest NMAE with sampling locations of more than 40 and improves PSNR faster than the other three algorithms. When there is strong noise of 30 dB and 20 dB, the SL0 algorithm and the TwIST algorithm have smaller PSNRs and larger NMAEs, but the ReSL0 algorithm can still achieve good reconstruction results with sufficient measurements. In other words, the ReSL0 algorithm achieves good reconstruction of PAI images in noisy environments.

**Figure 6:**
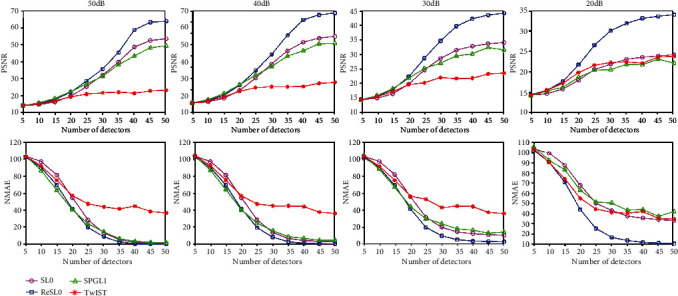
The PSNRs and NMAEs of reconstruction results of the blood vessel phantom with different number of sampling points. (a–d) The quantitative results of noisy observation with SNR = 50, 40, 30, and 20 dB, respectively.

Figure 6 provides the PSNR and NMAE tendency chart of the reconstruction results of the blood vessel phantom experiment with noisy observation. When the number of sampling locations is greater than 25, the ReSL0 method can achieve the largest PSNR and the smallest NMAE. These results are consistent with the results of the noiseless environment as shown in Table 2. However, the performance of the SL0 algorithm degrades rapidly due to the equality constraint in Eq. (8). According to the information gathered above, we may reach the conclusion that the ReSL0 algorithm can reconstruct high-quality PAI images regardless of noise. Besides, the SL0 algorithm that does not include noise regularization terms cannot be used in a strong noise environment. The inequality optimization constraint in Eq. (11) can provide the better performance in the noise environment.

### 3.3. In Vitro Experiments

**Figure 7:**
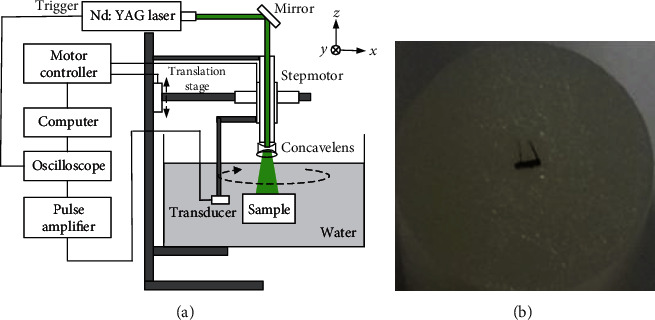
(a) The schematic diagram of PAI imaging system. (b) A photograph of the imaging sample.

Besides the numerical phantom experiments, the in vitro experiments were used to demonstrate the ReSL0 algorithm's performance. And the reconstruction results of the TwIST, SPGL1, and SL0 are also presented. A schematic diagram of the imaging system setup is displayed in Figure 7(a), where an experimental coordinate system [*x*, *y*, *z*] is also described. A Q-switched Nd:YAG pumped at 532 nm was used as the light source. The laser frequency is 10 Hz, and the pulse width is 6-10 ns. A focused piezoelectric transducer (Panametrics V309) with a central frequency of 5 MHz was controlled by a precision stepper motor for photoacoustic signal acquisition. The rotation radius of the transducer is 40 mm, and the rotation step size is 2°. At each sampling location, the photoacoustic signals were first amplified by a signal amplifier, then captured and averaged 30 times by a Tektronix MSO4000B mixed signal oscilloscope, and finally transmitted to the computer for signal processing and imaging.

The imaging sample used in the experiment is a gelatin cylinder with one graphite rod and two hairs as optical absorbers.  Figure 7(b) is the photograph of the sample. The radius of the gelatin cylinder is 13 mm. The diameter of graphite rod is 0.5 mm, and the length of hair is 4 mm. In the phantom experiment, 60-view data and 90-view data are selected for reconstruction. The images are constructed by the TwIST, SPGL1, SL0, and ReSL0 algorithms, respectively.

**Figure 8:**
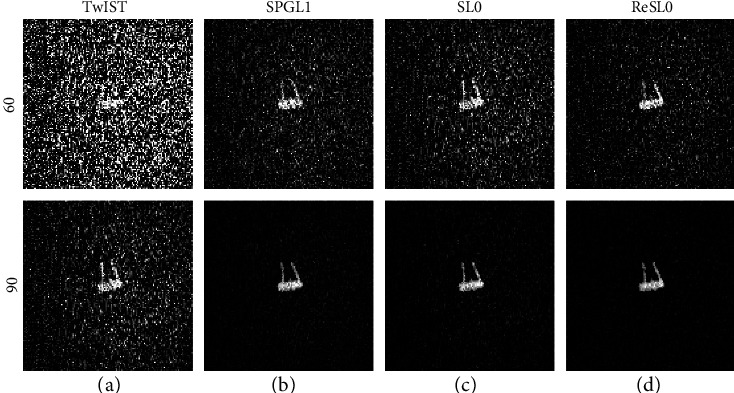
The reconstruction results from 60-view and 90-view experiment data, respectively. (a–d) Results of TwIST, SPGL1, SL0, and ReSL0 individually.

The in vitro experiment results are displayed in Figure 8. The first and the second row show the reconstruction results from 60-view and 90-view experiment data. As can be seen from the first row of Figure 8, there will be a lot of noise in the reconstructed image and some low-contrast features will disappear when using 60-view experiment data. When the sampling data is sufficient, such as 90-view data, all four algorithms are able to construct good quality images. And the sizes and locations of the optical absorbers are well reconstructed. However, the resolution of reconstructed images using TwIST and SL0 algorithms is not as good as SPGL1 and ReSL0 algorithms. By comparing the results of numerical experiments and in vitro experiments, we can conclude that the quality of reconstructed images in vitro experiment is not as good as that of numerical experiments. Therefore, we need to collect more data to achieve exact reconstruction.

## 4. Conclusion

In this paper, we carried out and estimated a L_0_ norm-based ReSL0 algorithm for the PAI. The main motivation of it is to replace the equality constraint with inequality constraint that allows some errors and make use of a regularization parameter to achieve a balance between the sparsity and the residual of objective function. The effectiveness and universality of this algorithm are demonstrated through numerical and in vitro experiments. Both visual inspection and quantitative measure comparisons have manifested that the ReSL0 algorithm can reconstruct better images than L1 norm and TV norm-based CS algorithms. Furthermore, the ReSL0 algorithm has similar computational efficiency to the SL0 algorithm and at the same time has better immunity to noise. Finally, the ReSL0 algorithm can significantly reduce the number of ultrasonic sensors and scanning time required to reconstruct high-quality PAI images.

## Figures and Tables

**Algorithm 1:**
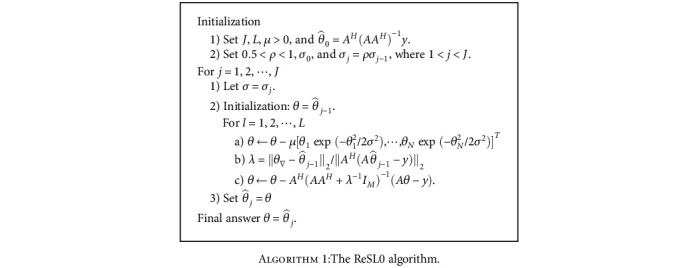
The ReSL0 algorithm.

**Table 1:** Numerical results of reconstructed images.

Experimental	CPU time (seconds)				PSNR (dB)				NMAE			
Positions	TwIST	SPG	SL0	ReSL0	TwIST	SPG	SL0	ReSL0	TwIST	SPG	SL0	ReSL0
5	0.69	11.76	0.52	0.77	11.8	11.93	11.95	12.11	104.93	103.34	103.08	101.25
10	1.1	12.3	1.09	1.45	12.57	13.27	12.65	13.21	96.01	88.58	95.17	89.15
15	1.93	22.76	1.86	2.18	13.34	14.78	14.14	14.68	87.87	74.48	80.12	75.31
20	2.58	26.07	2.51	2.81	14.65	16.04	15.6	16.11	75.53	64.35	67.77	63.84
25	3.32	28.67	3.36	3.71	16.01	17.34	16.8	17.2	64.61	55.47	59	56.37
30	3.9	51.92	4.04	4.45	17.03	18.94	18.05	18.94	57.46	46.09	51.1	43.99
35	2.65	46.86	4.77	5.14	18.35	21.45	20.22	21.66	49.34	33.7	39.79	31.63
40	3.59	30.22	5.41	5.77	19.7	30.23	23.9	30.23	42.26	12.57	26.06	11.66
45	4.04	29.03	6.44	6.8	21.3	42.66	40.31	60.93	35.16	3.01	3.94	0.37
50	3.08	28.8	7.11	7.61	24.5	61.36	83.65	113.5	24.3	0.35	0.03	0.001
Average	2.69	28.84	3.71	4.07	16.93	24.80	25.73	31.86	63.75	48.19	52.61	47.36

**Table 2:** Numerical results of reconstructed images.

Experimental	CPU time (seconds)				PSNR (dB)				NMAE			
Positions	TwIST	SPG	SL0	ReSL0	TwIST	SPG	SL0	ReSL0	TwIST	SPG	SL0	ReSL0
5	0.56	6.64	0.49	0.72	14.51	14.28	14.3	14.41	100.73	103.39	103.24	101.96
10	1.19	17.67	1.02	1.44	16.06	15.86	14.87	15.52	84.25	86.27	96.61	89.68
15	1.65	21.68	1.76	2.05	19.94	18.52	16.39	17.79	53.93	63.5	81.14	69.09
20	2.53	25.83	2.37	2.82	34.14	22.43	19.9	22.24	10.51	40.48	54.16	41.38
25	2.18	19.71	3.29	3.5	37.89	27.01	25.51	28.72	6.83	23.9	28.4	19.63
30	2.65	28.07	3.87	4.14	41.42	31.46	32.24	35.79	4.54	14.31	13.08	8.7
35	2.65	34.98	4.47	4.83	43.35	39.04	40.33	45.64	3.64	5.98	5.16	2.8
40	2.73	18.22	5.22	5.77	45.4	47.6	53.94	65.24	2.88	2.23	1.08	0.29
45	2.29	21.71	5.85	6.47	46.47	65.3	87.61	112.95	2.54	0.29	0.02	0.001
50	2.48	8.45	6.81	7.16	47.88	81.64	115.34	115.71	2.16	0.04	0.001	0.001
Average	2.09	20.3	3.52	3.89	34.71	36.31	42.04	47.40	27.2	34.04	38.29	33.35

**Table 3:** Numerical results of reconstructed images.

Experimental	CPU time (seconds)				PSNR (dB)				NMAE			
Positions	TwIST	SPG	SL0	ReSL0	TwIST	SPG	SL0	ReSL0	TwIST	SPG	SL0	ReSL0
5	0.37	8.04	0.45	0.74	14.58	14.49	14.81	14.95	96.87	97.58	97.89	93.45
10	0.97	12.47	1.08	1.5	14.76	15.46	15.4	15.79	88.17	84.36	85.86	80.16
15	1.48	21.62	1.78	2.08	15.43	16.38	16.29	16.67	78.49	74.94	74.68	68.14
20	2.15	31.96	2.55	2.89	16.27	17.34	17.18	17.73	68.62	63.55	62.05	54.09
25	2.83	37.57	3.21	3.48	17	18.57	18.18	18.92	60.94	51.36	49.37	43.06
30	3.36	49.94	4.15	4.48	17.99	20.55	19.98	21.02	51.78	37.85	34.52	29.41
35	3.17	26.03	4.73	5.15	18.98	22.43	22.26	23.47	44.38	27.93	22.88	18.97
40	3.03	32.1	5.5	5.85	20.2	25.01	25.52	26.49	37.09	19.32	14.04	11.8
45	2.92	52.29	6.18	6.82	21.64	27.24	27.76	29.12	30.51	13.31	9.1	6.96
50	3.43	53.99	6.75	7.14	23.49	30.16	30.99	32.99	23.54	8.4	5.43	3.91
Average	2.37	32.6	3.64	4.01	18.03	20.76	20.84	21.72	58.04	47.86	45.58	41

## Data Availability

The data that support the findings of this study are available from the corresponding author upon reasonable request.
